# Correction: Children’s right to play in Chilean hospitals: A forgotten right?—A qualitative study protocol

**DOI:** 10.1371/journal.pone.0334056

**Published:** 2025-10-09

**Authors:** Aleksandra Głos, Alejandra Santana López, Ivonne Vargas Celis, Lilian Sanhueza Díaz, Constanza Quezada

There are errors in the author affiliations. The correct affiliations are as follows:

Aleksandra Głos^1^, Alejandra Santana López^2^, Ivonne Vargas Celis^2^, Lilian Sanhueza Díaz^4^, Constanza Quezada^2,3^

**1** Faculty of Social, Legal and Humanities Sciences, Gabriela Mistral University, Santiago de Chile, Chile, **2** School of Nursing, Faculty of Medicine, Pontifical Catholic University of Chile, Santiago de Chile, Chile, **3** Faculty of Social, Legal and Humanities Sciences, Gabriela Mistral University, Santiago de Chile, Chile, **4** Faculty of Social, Legal and Humanities Sciences, Catholic University of Temuco, Temuco, Chile
