# Supplementary material for: Children’s right to play in Chilean hospitals: A forgotten right?—A qualitative study protocol
Source: PLoS One. 2025 May 12;20(5):e0316925. doi: 10.1371/journal.pone.0316925 (PMC12068643; doi:10.1371/journal.pone.0316925)
Supplement: S1 File — (DOCX) [file pone.0316925.s001.docx]

**Pauta de entrevistas semi-estructuradas: actores claves**

Contexto: *buscamos conocer sus opiniones sobre la importancia del juego de los niños y niñas en el ámbito hospitalario y las distintas oportunidades para jugar que tienen en su institución.*

1. **Pregunta inicial**
2. Para empezar, ¿cuál es su cargo y como se relaciona con el juego en la institución de salud donde trabaja? o/y Nos podría contar de qué manera su experiencia laboral se relaciona con el juego en el hospital/institución?
3. **Concepto y la importancia del juego para NNA en hospital y al final para adultos que trabajan con ellos.**
4. ¿Cuál es la importancia del juego?
   1. Desde su experiencia laboral, ¿es importante el juego en ámbito hospitalario? ¿Por qué sí? ¿Por qué no?
5. ¿Cuál es la importancia del juego para el bienestar y salud mental de los NNA?
6. ¿Cuál es la importancia de juego para los adultos que trabajan con NNA en el ámbito hospitalario?
   1. ¿En qué juego les puede ayudar?
   2. Es importante juego para el bienestar / salud mental de adultos que trabajan con NNA (porque)? ¿Cuál es su experiencia al respecto?
7. En su práctica profesional, ¿hubo alguna experiencia/ algún caso (por ejemplo, situaciones particulares con pacientes pediátricos) que le hiciera entender la importancia del juego para niños hospitalizados?
8. En su práctica profesional, ¿hubo alguna experiencia/algún caso (por ejemplo, situaciones particulares con pacientes pediátricos) que le hiciera experimentar el “milagro” del juego o magia del juego” (que el juego cambió el bienestar de un niño, le hizo abrirse, le mejoró la salud/salud mental/bienestar en hospital? (una historia relacionada con juego que le impactó como profesional)
9. **El derecho a jugar**
10. El juego es reconocido por la Convención sobre los Derechos del Niño, En su institución, ¿se entiende el juego como un derecho de NNA y se intenta proporcionarlo?
    1. ¿Cree que la importancia del juego para los niños y adolescentes se comprende en general? ¿piensa que el juego es valorado en la institución? ¿de qué manera se expresa esa valoración? ¿Y de parte de otros/as profesionales del hospital?
11. ¿Existe alguna sala de juegos especial para niños? Si este no es el caso, ¿hay otro lugar donde los niños puedan jugar? Y si esto es así, ¿juegan? ¿Si usted ve los NNA jugando?
    1. Otras preguntas para profundizar: ¿quién lo organiza? ¿Cuáles son las normas? ¿cómo se financia?) ¿Hay un especialista/un equipo que facilita juego?
12. ¿Hay política de juego? ¿Quién la desarrolló y cuál es su objetivo? (Solo para jefes) *Si es necesario especificar: ¿Es decir: un documento interno que p.ej. garantice el derecho al juego y defina las condiciones para jugar en las salas de juego: cuándo, cómo y con quién se permite jugar (por ejemplo, ¿se permite a los padres jugar con los pacientes ingresados?)*
13. Desde su experiencia, ¿de quién diría que depende que pueda implementarse más el derecho al juego? (salud pública, recursos, administrativos, personal de salud, ideologías). Preguntar solamente si el contexto lo posibilita.
14. **Juego libre (explicación: *juego en el que los niños están a cargo del juego, establecen sus propias reglas, crean espontáneamente sus propios mundos, en contraste con los juegos prediseñadas y dirigidas por adultos que sirven a objetivos definidos por adultos*.**
15. ¿Pueden los niños jugar libremente en el espacio designado para el juego o el juego está organizado y dirigido por los adultos?
16. ¿Le parece importante que los NNA jueguen libremente? ¿Por qué?
17. ¿Cómo se promueve que los NNA jueguen libremente en el hospital?

1. ¿Si usted ve los NNA jugando libremente (p.ej. en sus salas, en corredores u otros lugares)? ¿En que juegan los NNA? Si no juegan, qué obstáculos hay (p.ej. seguridad, ¿no sienten derecho)?

**V. Juego médico**

1. ¿Cómo emplean el juego los profesionales sanitarios cuando trabajan con niños? ¿Parecen comprender la importancia del juego en su práctica?

a) Especificación, si es necesario: por ejemplo, una enfermera u otro profesional que usa el juego para preparar a los niños para procedimientos dolorosos o aterradores, como resonancias magnéticas o inyecciones, o médicos que usan el juego al realizar un procedimiento o explicar la afección médica. ç

b) ¿Han recibido formación, cuentan con el apoyo de personal adicional?

1. En su práctica profesional, ¿hubo algún caso (algunas situaciones particulares con pacientes pediátricos) que le hiciera comprender la importancia de este tipo de juego, por ejemplo, como estrategia de información, rehabilitación, terapia, etc.)? ¿Podría contarnos más al respecto?
2. A veces se dice que «el juego es el idioma» de los niños. ¿Considera que el juego puede ser una forma más eficaz de comunicación con los niños? ¿Cómo puede el juego facilitar la comunicación con los niños? (Pida algunos ejemplos).
3. El juego no es valorado de la misma manera por todos los adultos o profesionales de la salud. En su práctica, ¿ha observado interacciones de profesionales médicos con niños que promueven o restringen el juego? ¿En qué sentido? ¿Cómo sucede eso? ¿Por qué?

**VI. El futuro de juego**

1. ¿Qué factores favorecen el juego en los hospitales?
2. ¿Qué factores limitan el juego en los hospitales?
3. ¿Si estuviera en sus manos hacer algunos cambios en su institución para que los NNA puedan disfrutar más de juego, que haría? Se puede soñar….

**VII. Preguntas finales**

1. ¿Desea añadir algo más que no preguntamos y qué le parece importante de añadir?
2. ¿Hay algo que quiera preguntarme?
