# Supplementary material for: Children’s right to play in Chilean hospitals: A forgotten right?—A qualitative study protocol
Source: PLoS One. 2025 May 12;20(5):e0316925. doi: 10.1371/journal.pone.0316925 (PMC12068643; doi:10.1371/journal.pone.0316925)
Supplement: S2 File — (PDF) [file pone.0316925.s002.pdf]

Pauta de Observación - Espacio de Juego

1. Para el registro:

El/la observador/a se ubicará en un lugar en que tenga vista panorámica de la situación a observar, correspondiente al lugar en que se ubicar el espacio destinado a los/as niños/as (sala de juegos o similar).

Se registrarán un tiempo máximo de XX minutos. Los que se registrarán en tramos de 5 minutos.

Ejemplo:

Hora de inicio: 9.45 hrs.

Hora de término: 10. 45 hrs.

Gráfica:

Se inserta un dibujo o fotografía panorámica de la situación a observar.

Tramo: 9.45 – 9.50:

Descripción de lo observado

Tramo: 9.50 a 9.55:

Descripción de lo observado:

(Así sucesivamente)

2. Para el análisis:

I. DESCRIPCIÓN DEL LUGAR PARA JUEGO *(si no hay espacio específico para juego, ir a sección III)*	
**Accesibilidad del lugar:**<br>- Lugar dentro del centro de salud en que se encuentra<br>- Indicaciones para llegar al lugar (se dispone de ellas, son claras, son precisas).<br>- Considera la posibilidad de acceso para NN con movilidad reducida (de qué manera)<br>- Posee vías de acceso y de salidas claras y seguras<br><br>Acceso (cómo llegar, accesible, visible)<br>¿Existen indicaciones claras y visibles para acceder al espacio de juego?<br>¿Cuenta con las medidas necesarias para acomodar a NNA con movilidad reducida?	

**Infraestructura, equipamiento y disposición del espacio**<br><br>Dimensiones del lugar (metros)<br>Organización del espacio (secciones o sub-ambientes) favoreciendo el desarrollo de juegos<br>Ventilación<br>Calefacción<br>Luminosidad (natural/ artificial)<br>Tipo de piso<br>Cercanía con servicios higiénicos<br>Mobiliario que facilita instancias de juego, acorde a niños y niñas<br><br>Infraestructura (tamaño, ventanas, puertas)<br>¿El espacio es físicamente seguro?<br>¿Las dimensiones del espacio que se observa favorece el juego como actividad?	
**Materiales, acondicionamiento y recursos disponibles para el juego:**<br>- El lugar dispone de juguetes, materiales lúdicos o materiales para favorecer la creatividad y el desarrollo sensorial de niños y niñas<br>- Se dispone de cuentos, libros de cartón o de estimulación sensorial.<br>- El lugar está decorado acorde a los intereses y estimulación	

infantil<br>- Se cuenta con equipamiento de juegos como mini toboganes, sube y baja, otros.<br>- Las juguetes y materiales son ajustados a diferentes grupos de NNA (género, edad, otro)<br><br>Ambientación (luz, mobiliario, decoración, otro)<br>¿El ambiente sensorial favorece el juego?	

Materiales disponibles (juegos, libros)<br>¿Se observan materiales como juegos o libros? ¿Los materiales disponibles apoyan el juego como actividad?	
**Organización del espacio**<br>Modalidad en que está organizado el espacio<br>Disposición del espacio para jugar<br>Disposición del espacio para otras actividades<br>Distinciones de disposición de la sala según características de los niños y niñas (género, edad, otro).<br><br>Disposición del espacio (orden, intencionalidad)<br>¿La disposición y organización del espacio de juego invita a jugar?	
**Usabilidad de la sala**<br>Uso dado a la sala<br>Tiempos de uso dado a la sala<br><br>Usos de la sala (uso exclusivo para el juego o no)	
Grupos a los cuales se orienta la sala (género, edad, otro)	
***II.* DINÁMICAS DEL JUEGO EN LA SALA DE JUEGOS (espacio)**	

**Objetos elegidos por los niños y niñas para jugar**<br>Juguetes elegidos para juego individual<br>Juguetes elegidos para juegos con otros niños<br>Juguetes elegidos para juegos con adultos<br>Otros objetos elegidos (libros, materiales, otros) para juego individual<br>Otros objetos elegidos (libros, materiales, otros) para juego con otros niños<br>Otros objetos elegidos (libros, materiales, otros) para juego con adultos<br><br>¿Qué tipos de juguetes hay?<br>¿Qué tipos de juegos hay? ¿En qué consisten?	

¿Con qué juegan los niños?<br><br>¿Juegan en grupo o solos?<br>**Relaciones establecidas en el contexto de juego**<br>Niños juegan solos<br>Niños juegan con otros niños<br>Niños juegan con adultos familiares<br>Niños juegan con niños y adultos simultáneamente<br>Niños juegan con niños y adultos agentes a cargo de actividades de la sala	
**Características del juego**<br>Grado de estructuración del juego<br>Juego libre<br>Juego organizado<br>Descripción del juego<br><br>¿Podemos calificar la observación dentro del juego libre o más bien como juego organizado para niños? (especificación juego libre, ir a sección III)	***ELECCIÓN: ¿Se puede elegir entre varios juegos? ¿los niños proponen/inventan sus propios juegos?***
**III. DINÁMICAS DE JUEGO EN OTROS ESPACIOS**	
¿Qué tipos de juego hay? ¿En qué consisten?	

¿Con qué juegan los niños?<br>¿Juegan en grupo o solos?	

¿Podemos calificar la observación dentro del juego libre o más bien como entretenimiento organizado para niños? (especificación pauta III juego libre)	*ELECCIÓN: ¿Se puede elegir entre varios juegos/juguetes? ¿los niños proponen sus propios juegos?*
	VOLUNTARIO: ¿la participación es voluntaria? ¿Y es voluntario retirarse del juego?
	CONTROL: ¿Los adultos dirigen y controlan el juego? ¿Los niños controlan el juego (y los adultos sólo lo facilitan)?
	FINALIDAD: ¿Tiene el juego una finalidad (por ejemplo, educativa, terapéutica, otra) o es sólo para divertirse?
