# Supplementary material for: Children’s right to play in Chilean hospitals: A forgotten right?—A qualitative study protocol
Source: PLoS One. 2025 May 12;20(5):e0316925. doi: 10.1371/journal.pone.0316925 (PMC12068643; doi:10.1371/journal.pone.0316925)
Supplement: S3 File — (PDF) [file pone.0316925.s003.pdf]

Pauta de auto evaluación

El derecho al juego es también un derecho humano fundamental y específico, reconocido por la comunidad internacional y recogido en la Convención de las Naciones Unidas sobre los Derechos del Niño (UN CDN, 1989) en su artículo 31, ratificado por casi todos los países (a excepción de EE. UU.), incluido Chile.
Este es un cuestionario traducido de un documento elaborado por la OMS. El objetivo de este es que usted responda de acuerdo con la realidad de institución. Responda a cada una de las preguntas según corresponda.

**Elementos a evaluar**	**Sí**	**No**
1. La evidencia sugiere que los niños, niñas y adolescentes (en adelante, NNA) de todas las edades tiene oportunidades para el juego y el ocio de acuerdo con su edad y preferencias (por ejemplo, tanto niños y niñas, como adolescentes)		
2. El hospital provee de otras actividades de apoyo como grupos de payasos, música, arte y/o terapia con animales o servicios por el estilo		
3. Todos los doctores y enfermeras utilizan el juego en el cuidado terapéutico.		
4. Las opiniones de los NNA se consultaron durante la planificación de la sala de juegos o han sido consultadas en etapas posteriores sobre lo apropiado del espacio y cómo mejorarlo.		
5. La evidencia recolectada de los NNA y sus padres muestran que están satisfechos con la disponibilidad de servicios de juego.		
6. El hospital promueve la investigación sobre los beneficios de usar el juego durante el cuidado terapéutico u otras actividades de apoyo, las cuales son publicadas y compartidas con audiencias más amplias.		
7. Existe una política de hospital que garantiza el derecho de los NNA a jugar.		
8. Existe una sala de juego equipada de manera apropiada.		
9. Existen especialistas en juego para apoyar a los niños mientras juegan.		
10. Cada NNA es motivado y apoyado a jugar, incluso si no pueden abandonar su cama.		
11. La mayoría de los doctores y enfermeras han recibido capacitaciones sobre cómo utilizar el juego en el cuidado terapéutico y lo aplican.		
12. Existe un colegio hospitalario, profesor/a capacitado/a u otro sistema que permite que los niños continúen con su educación mientras se encuentran en el hospital.		
13. Una política de juego se encuentra en desarrollo		

Pauta de auto evaluación

El derecho al juego es también un derecho humano fundamental y específico, reconocido por la comunidad internacional y recogido en la Convención de las Naciones Unidas sobre los Derechos del Niño (UN CDN, 1989) en su artículo 31, ratificado por casi todos los países (a excepción de EE. UU.), incluido Chile.

Este es un cuestionario traducido de un documento elaborado por la OMS. El objetivo de este es que usted responda de acuerdo con la realidad de institución. Responda a cada una de las preguntas según corresponda.

14. No existe una sala de juego para NNA, pero sí un espacio donde pueden ir y jugar con otros NNA.		
15. El juego se utiliza en el cuidado terapéutico por algunos profesionales que han participado en capacitaciones de ese estilo.		
16. Existen algunas posibilidades para que NNA continúen su educación mientras se encuentran en el hospital.		
17. No existen políticas internas que garanticen el derecho a jugar de NNA.		
18. No existe sala de juegos.		
19. No existen miembros del equipo especialistas en el juego (por ejemplo, especialistas de juego).		
20. El juego no se utiliza en el cuidado terapéutico (por ejemplo, para estimular el desarrollo, en la preparación para procedimientos, como distracción o apoyo para que los NNA puedan expresar sus sentimientos)		
21. No existen posibilidades para que NNA continúen su educación durante su estadía en el hospital (por ejemplo, a través de colegios hospitalarios, profesores capacitados u otro tipo de sistema)		
