# Supplementary material for: Children’s right to play in Chilean hospitals: A forgotten right?—A qualitative study protocol
Source: PLoS One. 2025 May 12;20(5):e0316925. doi: 10.1371/journal.pone.0316925 (PMC12068643; doi:10.1371/journal.pone.0316925)
Supplement: Attachment — Submitted filename: Response to Reviewers.docx [file pone.0316925.s005.docx]

Dear Editors,

Thank you for giving us the opportunity to submit the revised version of the study protocol: “Children’s Right to Play in Chilean Hospitals. A Forgotten Right? – A Qualitative Study Protocol.” We are very grateful for the very insightful and detailed reviews we received. We found the comments very accurate and incorporated all the suggestions made by Reviewer 3 (see: tracked changes mode (simple revisions) and highlighted). As requested, we also attach point-by-point responses to Reviewer 3 with all the requested changes included.

**Comment 1:** Please ensure that your manuscript meets PLOS ONE's style requirements, including those for file naming.

**Response:** Thank you, we have corrected the naming style, as well as the sections and figures formatting according to PLOS ONE’s guideline.

**Comment 2:** Thank you for stating the following financial disclosure:

“ANID, Fondo Nacional de Desarrollo Científico y Tecnológico: Fondecyt de Iniciación en Investigación 2023: From patients to citizens. A study on narrative solidarity in bioethics. Grant Number: 11230025

Please state what role the funders took in the study.  If the funders had no role, please state: "The funders had no role in study design, data collection and analysis, decision to publish, or preparation of the manuscript." If this statement is not correct you must amend it as needed. Please include this amended Role of Funder statement in your cover letter; we will change the online submission form on your behalf.

**Response:** Indeed, the funders had no role in study design, data collection and analysis, decision to publish, nor preparation of the manuscript and we have amended the role of the funder in the cover letter, as requested.

**Comment 3:** Please include your full ethics statement in the ‘Methods’ section of your manuscript file. In your statement, please include the full name of the IRB or ethics committee who approved or waived your study, as well as whether or not you obtained informed written or verbal consent. If consent was waived for your study, please include this information in your statement as well

**Response**: The study was approved by the *UC Health Sciences Scientific Ethics Committee* and the written resolution certificate was obtained (ID: 231218019), which we included in the ethics statement in the Methods section (p. 14).

**Comment 4:** Please review your reference list to ensure that it is complete and correct. If you have cited papers that have been retracted, please include the rationale for doing so in the manuscript text, or remove these references and replace them with relevant current references. Any changes to the reference list should be mentioned in the rebuttal letter that accompanies your revised manuscript. If you need to cite a retracted article, indicate the article’s retracted status in the References list and also include a citation and full reference for the retraction notice

**Response:** Thank you for pointing that out. Indeed, we removed a passage on the gender dimensions of play, which in this study we examine only to the minimal extent (while observing equipment of playrooms and checking whether toys and materials for different ages, abilities and gender groups are provided). While deleting the short passage we overlooked the withdrawing of references, which we have now done. It regards the following positions:

While including Reviewer 3’s suggestions to the manuscript, we also added the following positions to the literature:

American Academy of Pediatrics (AAP). Committee on hospital care: Care of children in hospitals. Evanston, IL; 1960.

Canadian Paediatric Society. Position statement: Child life in health care settings. Ottawa, Canada: Canadian Paediatric Society; 1978.

Humphreys C, LeBlanc CK. Promoting Resilience in Paediatric Health Care: The Role of the Child Life Specialist. Child and Adolescent Resilience Within Medical Contexts, In: C DeMichelis & M Ferrari, editors. Child and adolescent resilience within medical contexts: Integrating research and practice. Switzerland: Springer International Publishing/Springer Nature; 2016, pp. 153–173.

American Academy of Pediatrics (AAP), Child Life Council and Committee on Hospital Care.Child Life Services. Pediatrics. 2006 Oct; 118(4): 1757–1763. 10.1542/peds.2006-1941

American Academy of Pediatrics, Committee on Hospital Care and Child Life Council. Child life services. Pediatrics, 2021; 147(1): e2020040261.

Rosenblatt A, Pederson R, Davis-Sandfoss T, Irwin L, Mitsos R, Manworren R. Child life specialist practice and utilization across health care: a scoping review protocol. JBI Evidence Synthesis. 2022 Aug 30;21(2):407–13.

Flick, U. Doing Interview Research: The Essential How to Guide. Thousand Oaks, CA: SAGE Publishing; 2022.

Emerson, R. M., et al. Writing Ethnographic Fieldnotes. 2nd ed. Chicago: The University of Chicago Press; 2011.

Ciesielska M, Boström KW, Öhlander M. Observation Methods. In: M. Ciesielska, D. Jemielniak, editors. Qualitative Methodologies in Organization Studies. Cham: Palgrave Macmillan; 2018:33–52.

Dear Reviewer 3,

Thank you very much for reviewing our work and for providing such insightful, detailed comments. We found the advice friendly and the criticism constructive. According to your suggestions, we made the following changes:

I would like to highlight that the proposal has significant potential to contribute to the advancement of knowledge and its implementation in developing countries such as Chile. The proposal is well-structured, clear, and addresses a relevant topic with a robust scientific approach. Additionally, it objectively presents the theoretical context of the research, emphasizing the importance of the right to play for hospitalized children and justifying the relevance of the study in the Chilean context. However, a few aspects could be improved or clarified to further strengthen the quality of the proposed research.

- **Comment: 1**. *In the introduction, it is pertinent to highlight the reality of developed countries, such as the United States and Canada, which differ significantly from South American countries due to the presence of professionals known as Child Life Specialists. These professionals focus on using play to promote the well-being of hospitalized children. Comparing these distinct realities could further emphasize the relevance of the proposed study, demonstrating that, regardless of the existence of a professional dedicated to this role, play should be recognized and promoted as a fundamental right of every child.*

**Response:**

Thank you for this comment. Indeed, the idea of the study emerged from the comparison between the Chilean reality and the far-reaching implementation of children's right to play in hospitals in developed countries, where there is not only the practice but also a longtime, ongoing academic discussion on this topic, which is still very rare in the Latin American context. We are very grateful for this comment, as it is indeed important to express this comparative perspective more clearly. Moreover, this comparison will help us to build argumentation to promote the greater recognition of children's right to play in hospitals, which we intend to do through this research (and its follow-up). The long experience of the United States and Canada and the evidence-based effectiveness of child life specialist practice can, mutatis mutandis, serve as a model for other countries.

**Changes in the manuscript: We added the following fragment (please check p. 3-4)**

It is worth mentioning that many paediatric hospitals and health care facilities in developed countries employ certified professionals to provide psychosocial support and interventions for children and families. For example, in the United States, as early as 1960, the American Academy of Paediatrics (AAP)^24^ recommended that all paediatric wards should have a playroom equipped with appropriate materials such as games, toys and books, while the Canadian Paediatric Society in 1978^25^ advocated the employment of child life specialists to meet the psychosocial needs of hospitalised children. The acknowledgement of play was a significant step in recognizing the psychosocial needs of hospitalized children and improving patient care^26^. It resulted in the foundation of the child life profession, which is now present in the majority of paediatric hospitals in the US and Canada^27^. The child life specialists are certified professionals who use play and other psychosocial interventions to promote children’s well-being and minimize the adverse effects of hospitalization for young patients. There is consistent evidence that child life services improve quality and outcomes in paediatric care as well as the patient and family experience ^28,29^. This comparative perspective very clearly demonstrates the need to explore how developing countries such as Chile fulfil their obligations under UN CRC to provide play opportunities to hospitalized children, and what kind of obstacles they encounter along the way. As the study aims to identify factors that promote and limit the provision of this right, as well as collect health professionals’ experience in that respect, the data gathered might help to advocate for the greater implementation of children’s right to play in the context of limited resources.

- **Comment 2.** Although the project defines free play, chosen and directed by children, as a guiding principle of the process, it is important to emphasize that playful interactions between children and healthcare professionals are also valuable sources of data. These interactions can either promote or restrict play, depending on the actions of the professionals. Including an analysis in the interviews regarding how professionals approach play and their attitudes toward playful activities is insightful, but it could be further enriched if this analysis is also incorporated into the observation phase. For example, observing situations where professionals encourage or inhibit play (due to lack of time, perceptions of the value of play, or institutional factors) could reveal important dynamics. It is suggested to integrate these interactions into the observation process, considering the comparison between the narratives in the interviews and the actions observed in the environment, which are not always aligned.

**Response:** That’s a very astute remark. Our focus on free play is due to two main reasons; first is the fact that the intrinsic, non-instrumental (albeit with countless benefits) value of play is less recognized in the, by definition, utility-oriented hospital setting in general; secondly, it captures the fact that play is also the right of children. However, it is very true that medical play is not less important, as it has multiple benefits of reducing children's anxiety before the procedures, establishing a trusting relationship with healthcare providers and preventing medical trauma, among others. It is also true that professionals’ attitude has an enormous impact on play provision, and it may indeed “encourage or inhibit” play. Health professionals might not only engage (or not) in the medical play with children but also promote or inhibit their free play. We fully agree that it would be very valuable to observe these interactions and attitudes in clinical settings. Unfortunately, we are not able to include this aspect in the observation guidelines at this stage because we cannot yet (in this study) observe children, having to limit ourselves to observation of the spaces (playrooms, if they exist or other play spaces, their design, equipment and other aspects). This is because hospitals in Chile are very close, restricted institutions, adopting strict measures of child protection and security. Additionally, to the evaluation by the Ethics Committee of our university, the project had to go through the Ethics Committee of each institution (two of which were already obtained, additionally to two exemptions). As this is the first, exploratory, diagnostic study on the topic, we have decided to establish our relations with the hospitals in a step-by-step manner. In this study, we want to let ourselves be known to the hospitals through our good work and ethical conduct, so that in the second step (and the second study) we are also permitted to interview children and observe their interactions with medical professionals during play. Children are the main protagonist of our study, and it is an undeniable limitation that we cannot include them at this stage. However, the study is part of a larger project and a long-term commitment of the research team, so in the next study we will incorporate this helpful suggestion of the Reviewer to a greater extent. What we have been able to do at this stage to honor this suggestion is to make the following changes:

1)	We included more questions about the medical play and professional interaction dynamics of children in the semi-structured questionnaire. As the study design includes interviews with a diverse group of professionals (both of medical and non-medical specialization), we can get a broad picture of medical play, as well as its beneficial and inhibiting factors.

2)	We also explicitly addressed this particular limitation in the Study Limitations section.

**Changes in the manuscript and supplementary files:**

Ad. 1. Interview guide

We added additional questions to our semi-structured interview guide (point 5).

V. Medical play

1. How do healthcare professionals use play when working with children? Do they seem to understand the importance of play in their practice?

a)	Specification, if necessary: for example, a nurse or other professional using play to prepare children for painful or frightening procedures, such as MRIs or injections or doctors using play when performing a procedure or explaining the medical condition.

b) Have they received training, are they supported by additional staff?

2. In your professional practice, were there any cases (some particular situations with paediatric patients) that made you understand the importance of this type of play, for example, as an information strategy, rehabilitation, therapy, etc.)? Could you tell us more about it?

3. It is sometimes said that ‘play is a language’ of children. Do you consider that play can be a more effective form of communication with children? How can play facilitate communication with children? (Ask for some examples)

4. Play is not equally valued by all adults or health professionals. In your practice, have you observed the interactions of medical professionals with children that promote or restrict play? In what sense? How does that happen? Why?

**Ad. 2 Study Limitations (p. 17)**

We added the following passage (please check p. 17):

For the same reason, and due to the institutional limitations, we could not include observation of children at this research stage, limiting ourselves to the observation of play spaces and their equipment. However, both aspects will be addressed in future research.

- **Comment: 3**. The study's hypothesis is solid and addresses a real and potentially significant issue. Its approach offers the potential to reveal valuable insights into the disparities in upholding the right to play for hospitalized children in Chile.

Response: Thank you!

- **Comment 4.** The research setting is well-represented, and the selection of the four regions is appropriate to capture the geographical, socioeconomic, and cultural diversity of Chile, which may impact the guarantee of hospitalized children’s right to play.

Response: Thank you!

- **Comment 5**. The choice of a qualitative approach is appropriate as it allows for a deeper understanding of the factors influencing children’s opportunities to play in hospitals. This approach enables the exploration not only of the material and structural resources of healthcare institutions but also of the perceptions and decisions of healthcare professionals regarding play, which is essential for understanding practical barriers and opportunities. The focus on the different perspectives of healthcare professionals, including both medical and non-medical staff, is a relevant approach, as play can be influenced by various factors within the hospital. The comparison between interview narratives and observational

Response: Thank you!

- **Comment: 6**. Regarding the materials and methods, further clarification is needed on how the interviews will be conducted and the composition, roles, and qualifications of the research team. Will the research team be present in the various regions selected in the country? Are they residents of these regions? Will there be financial resources allocated for the team's travel? Who will conduct the interviews? Does the interviewer have expertise in qualitative interviews? Will there be training provided? Do they have any prior connection to the research setting? Additionally, it remains unclear whether the interviews will be conducted in person or remotely.

**Response:** Thank you for this comment. Yes, we did not explicitly touch upon these aspects so that could remain unclear. The research team resides in 2 of the chosen regions. As the longitudinal distances in Chile are very short, in contrast to latitudinal distances, conducting interviews will be possible in person in the 3rd, and via zoom in the 4^th^ (Northern Chile). Observation in each case will be conducted in person, as one of the students engaged in the project is originally a resident of the Arica y Parinacota region studying in Central Chile, the Metropolitan Region (the student will be remunerated and her travel cost will be covered). In any case, with the exception of the Arica y Parinacota region, we will combine in person and remote interviewing mode according to the preference and agenda of particular institutions and interviewees. The interviews will be conducted by members of the research team: four of whom (two social workers, one anthropologist, and one ethicist with qualitative experience) have several years of experience in doing qualitative interviews. The fifth member (a lawyer with interdisciplinary second degree) has a solid theoretical foundation and limited field experience (gained in the parallel ongoing project), and will therefore receive supplemental training by the best-qualified member of the research team. The whole team is totally impartial and is composed of academics having no prior connection to the research setting. We have completed the manuscript as regards the missing information, as can be seen below:

**Changes in the manuscript:**

a) **Sample, sample selection criteria, data saturation criteria (page 10)**

The research team had no prior contact with the selected institutions, which were chosen solely on the basis of their geographical location (being representative of each region) and characteristics (being a paediatric hospital or a general hospital with paediatric wards).

b) **Data collection (page 11)**

In the *first phase of data collection*, team members will conduct semi-structured interviews with key informants who have agreed to take part in the study. Interviews will be conducted by the members of the research team (composed of scholars of the following disciplines: two social workers, an ethicist, an anthropologist and a lawyer with an interdisciplinary second degree), four of whom have expertise and several years of experience in conducting qualitative interviews. The best-qualified member of the research team will train (according to the guidelines developed by Flick^87^ the fifth research team member, whose practical field experience is limited. Interviews will be conducted in person or via zoom depending on the preference of the interviewee and their health institutions. The Arica and Parinacota regions of northern Chile were important to include in the study, but interviews will only be conducted via zoom, as no member of the research team is a resident of this region. These will be recorded and then transcribed to ensure the accuracy of what was reported by the participants. This phase will be supplemented by an additional tool in the form of a self-assessment guide, which will be emailed to interview participants who have already agreed to it during the interview.

**Comment:** 7. The systematic approach to observation needs to be better described. Who will be the observer? Do they have experience with this method of data collection? Will training be provided on how to document the findings from the observations and ensure consistency among observers? How much time, in hours or days, is planned for the observations? Will they occur during different shifts? Will the individuals working in the observed settings be informed about this proposal?

If behaviors are being observed, I recommend consulting the Ethics Committee regarding the need to obtain informed consent (IC), even if prior approval has been granted. When observing behaviors, particularly in a context involving interactions with children, it is essential to ensure that informed consent is obtained. While the proposed non-participant observation might involve minimal or no interaction with individuals, it still requires informed consent, especially given the vulnerability of the child population.

**Response:** Thank you for pointing that out. We have now better described the approach to observation addressing each of the posed questions. The observation will be carried out by a group of four advanced anthropology students (two of whom will be writing a masters thesis related to the project). Each member of the team has prior experience in conducting field observations in research projects, as this was one of the criteria for acceptance into this study. This work will be supervised by an anthropologist from the research team, who will provide field training in the observation process, taking notes and photographs (as the hospitals have given permission to photograph the playroom when children are not inside), and transcribing the results. The anthropologist will supervise the process and evaluate each transcription of field notes based on the observation guidelines developed by the research team, indicating in each case their completion if necessary to ensure the quality and consistency of the results. They will take place at the time indicated by each institution (to ensure that children are not present). The observers will be introduced by the person designated to oversee the project at the hospital in question and introduced to those working in the institutions being observed. Each space will be observed at least three times at 1-hour intervals on different shifts and on at least two different days, and will be supplemented if necessary.

In the process of obtaining internal authorization from each hospital, we decided to refrain from observing the dynamics of play at this stage of the study, as the institutional willingness to grant permission to observe the spaces in which the hospitalized children were present varied. To ensure the consistency of results, we limited ourselves to observing the playroom space in each case (e.g., its physical characteristics, location, aesthetics, equipment and child-friendly design, usability) and other spaces where children can play (if any). The observation of these spaces will allow us to verify many aspects of the professional's narrative on the play opportunities provided to hospitalized children (e.g., whether the playroom and its equipment are independent of other therapeutic or educational purposes, whether it takes into account the needs of children of different ages and abilities etc.). After this, observation of the different dynamics of play (both as regards the free play and medical play) will be carried out and will comprise the subject of a separate study at the next stage of the project. (However, if in the course of the study we obtain the internal permission to extend our observation, we will apply for the necessary corrigendum to Ethics Committee certificate and request the informed consent and assent of parents and children involved).

**Changes in the manuscript (see page 11):**

In the second phase of data collection, non-participant observations of the play rooms and/or other play spaces in the hospitals will be carried out. The observation will be carried out by a group of four research assistants (advanced anthropology students), each with previous experience of field observation in research projects. This work will be supervised by an academic anthropologist from the research team, who will provide field training in the observation process, taking handwritten notes in a field notebook and photographs, and subsequent transcription of findings^88,89,95^. The anthropologist will supervise the process and evaluate each field note transcription based on the observation guidelines developed by the research team, indicating in each case their completion if necessary, to ensure the quality and consistency of the results. Observations will take place at the time indicated by each institution (to ensure that children are not present). Observers will be introduced by the person designated to look after the project at each hospital and presented to relevant individuals working in the institutions. In order to be more rigorous with the information collected, each space will be observed at least three times at 1-hour intervals on different days, and will be supplemented as necessary. The period of one hour of observation of the space allows us to see changes in it, for example if there are changes in light or if there some other than play activities conducted in the playroom^84^. In relation to the changes that the space may undergo, it was decided to go on different occasions (min. two different days).

- **Comment: 8**. I understand that the lack of information regarding how the research team will be composed, trained, and how the logistics for conducting observations in different hospitals will be handled is a significant gap in the proposal. In terms of resources, it would be crucial for the project to detail in the methodology whether there will be adequate financial support or logistical backing to ensure that the team has the necessary resources to travel, conduct observations, and collect data systematically. This would ensure the feasibility of the research and its potential for replication.

**Response:** Thank you for this remark. It is indeed necessary to assure the feasibility of this project conducted in different geographical locations in terms of financing and logistics. The study is part of the larger project financed by the leading Chilean finding agency (ANID, Chilean National Agency for Research and Development), so every student and member of the research group involved will be remunerated and all travel expenses will be covered. Moreover, the research team resides in two of the four regions. Due to the fact that the longitudinal distances in Chile are very short, one other region is at a very close proximity to the region of residence of the majority of the research team. As for the remaining region, the research assistant who will conduct observation is a resident of this region, while the interviews will be conducted via zoom. The need to describe better team composition and training was addressed in the response to previous comments (6 & 7).

**Changes in the manuscript (see page 12)**:

The study is funded by a grant from the Chilean National Agency for Research and Development (ANID), so every member of the project involved will be remunerated and all travel expenses will be covered.

Additionally:

The confirmation of funding was attached to the article submission as a supplementary file.

The changes in manuscript regarding the team composition and training were addressed in the response to previous comments (6 & 7).

- **Comment 9.** The proposal for analyzing the interviews and observation data is well-structured and follows a solid qualitative analysis approach. The use of a combination of deductive and inductive analysis is relevant, especially since the study is based on an in-depth and contextualized understanding of the phenomena, rather than seeking generalizations. Furthermore, the use of Atlas Ti software as a tool to facilitate the organization and coding of interviews is an effective choice. However, it would be interesting to provide more detail on how the integration between manual analysis and the software will be carried out. The idea of an interdisciplinary analysis also seems important, but could be more specified in terms of how different areas of expertise within the team will contribute to the analysis. Nevertheless, the proposal for triangulation is well-applied, relevant, and, in this context, provides clarity and depth to the research findings.

**Response**: Thank you for this comment. We have now described the auxiliary role of the software. Atlas Ti will be used for (1) document identification (transcription), (2) the transfer of codifications carried out manually and (3) the generation of code reports and related citations. The usage of the software will be instrumental, secondary and limited in application, in order to complement and systematize the interdisciplinary analysis conducted manually by the research team. To demonstrate the “division of labor” between the research team and the software, we have described the analysis process in more detail (see pages 12-13).

As regards the interdisciplinary triangulation, the research team consists of a lawyer with an interdisciplinary background (and a second degree in philosophy), an anthropologist, social workers and an ethicist. Therefore, the study will combine, respectively, legal knowledge of children's rights and, in particular, the child's right to play interpreted against a broader interdisciplinary background, with an anthropological perspective on play and its material culture and psychosocial conditions associated with the lives of children and their environments in the clinical setting. Researchers from different disciplines will be equally involved in the analysis and triangulation of the data, while being able to enrich specific parts of it with the expertise in their own disciplines.

**Changes made in the manuscript:**

a) **The semi-structured interviews analysis (page 12-13)**

The analysis will follow a six-phase process, as outlined by Braun and Clarke^90^. Specifically, the data will be transcribed by a professional who will sign the confidentiality agreement, and will then be anonymized by the principal investigator. All transcripts will be checked back against the audio recordings by the principal investigator. All researchers will code openly according to pre-established codes based on the research objectives, as well as new codes of an emergent nature that respond to the particularities of the contexts in which the data will be collected. The research team will hold an initial meeting to review the pre-established codes, agree on shared definitions and their precision parameters and potential attributes, in order to facilitate further analysis. Codes will be then collapsed or expanded, and the initial codebook will be produced by each author individually. The initial themes and subthemes developed by each author will then be triangulated, refined, discussed and conceptualized by the whole research team through team meetings, in which potential biases will be controlled and the interpretation of the data will be enhanced in a collective and interdisciplinary manner. The analysis and triangulation of data will be done by the research team manually. Additionally, the Atlas Ti software will be used for (1) document identification (transcription), (2) transfer of codifications carried out manually and (3) generation of code reports and related citations. The usage of the software will be instrumental, secondary and limited in application to complement and systematize in conjunction with the interdisciplinary analysis conducted manually by the research team. To enhance trustworthiness, we will apply Braun and Clarke’s 15-point checklist of criteria for thematic analysis to our study^90^.

b) **Triangulation of results (page 14)**

As the research team comprises a legal scholar with interdisciplinary background, an anthropologist, social workers and an ethicist, the study will combine, respectively, legal expertise in children’s rights and in particular children’s right to play, interpreted against the wider interdisciplinary background with anthropological perspectives on play and its material culture, and psychosocial conditions associated with the lives of children and their environments in clinical setting. Researchers from different disciplines will be equally involved in data analysis and triangulation (contributing from their perspective to a broad and comprehensive understanding of the research phenomenon), while being able to enrich the in-depth analysis of its different parts (for example, the presence of an anthropologist will ensure the quality of the ethnographic analysis of the place, a lawyer will ensure the correct analysis of the data in light of legal and public policy aspects, while social workers will be able to provide insights into the professional dynamics in health care and their impact on play, as well as enhance the understanding of children’s living conditions related to the hospital context).

- **Comment 10.** In summary, the study is promising and addresses a highly relevant topic with the potential to positively impact hospital practices related to the right to play. The suggestions presented aim to further strengthen the proposal, ensuring greater clarity and feasibility for its implementation.

**Response:** Thank you very much for this comment and for all the time and effort that you invested in preparing such a detailed and helpful review!
